# *TRPV6* Gene Mutation in a Dizygous Twin With Transient Neonatal Hyperparathyroidism

**DOI:** 10.1210/js.2018-00374

**Published:** 2019-01-03

**Authors:** Sumie Yamashita, Hiroshi Mizumoto, Hirotake Sawada, Yoshiro Suzuki, Daisuke Hata

**Affiliations:** 1 Department of Pediatrics, Kitano Hospital, Tazuke Kofukai Medical Research Institute, Osaka, Japan; 2 Division of Pediatrics, Department of Developmental and Urological-Reproductive Medicine, Faculty of Medicine, University of Miyazaki, Miyazaki, Japan; 3 Division of Cell Signaling, National Institute for Physiological Sciences, National Institutes of Natural Sciences, Okazaki, Japan

**Keywords:** maternal–fetal calcium transport, transient neonatal hyperparathyroidism, TRPV6

## Abstract

Maternal–fetal transport of calcium (Ca^2+^) is important for bone mineralization in fetal development. Insufficient Ca^2+^ transport causes transient neonatal hyperparathyroidism (TNHP). Transient receptor potential cation channel, subfamily V, member 6 (TRPV6), has been found to play an important role in the active transport of Ca^2+^ through the placenta. Recently, *TRPV6* gene was found to be the gene responsible for TNHP with severe skeletal undermineralization. To date, only seven cases of TNHP caused by *TRPV6* recessive mutations have been reported. We present a case of TNHP caused by *TRPV6* gene mutations. A female newborn was hospitalized because of respiratory distress. Marked undermineralization of the skeleton was observed in X-ray imaging. Laboratory examination revealed markedly high PTH and absence of hypercalcemia along with vitamin D deficiency. Her twin brother presented with almost no symptoms. Maternal laboratory findings indicated normocalcemia, but vitamin D deficiency with a high PTH level for the lactation period was observed. We initially diagnosed the patient as having secondary hyperparathyroidism because of maternal vitamin D deficiency. Nevertheless, the reasons underlying the discordant clinical manifestations between the twin siblings remained unclear. Our analysis of *TRPV6* gene clarified that the patient had compound heterozygote mutations, which were reported previously (p.Ile223Thr and p.Gly428Arg). Pathologic mutations in *TRPV6* gene were not detected in the other sibling. The clinical symptoms in the patient were transient: they resolved during infancy. TNHP caused by *TRPV6* gene mutations is a unique disease in terms of its transient pathology *in utero* and relief after birth.

The etiologies of transient neonatal hyperparathyroidism (TNHP) are heterogeneous. The major cause of TNHP is insufficient maternal–fetal calcium (Ca^2+^) transport. Transient receptor potential cation channel, subfamily V, member 6 (TRPV6), has been identified as one of the components of the apical Ca^2+^ entry pathway of intestine and placenta [1, 2]. In 2008, Suzuki *et al.* presented the first *in vivo* evidence that TRPV6 plays an important role in the active transport of Ca^2+^ through the placenta in an animal model [1]. Recently, Suzuki *et al.* [3] reported six patients with TNHP and severe skeletal undermineralization caused by *TRPV6* gene mutations. Subsequently, Burren *et al.* [4] reported an additional case. To date, only seven cases of TNHP caused by *TRPV6* mutations have been reported.

## 1. Case Report

**Figure 1.**
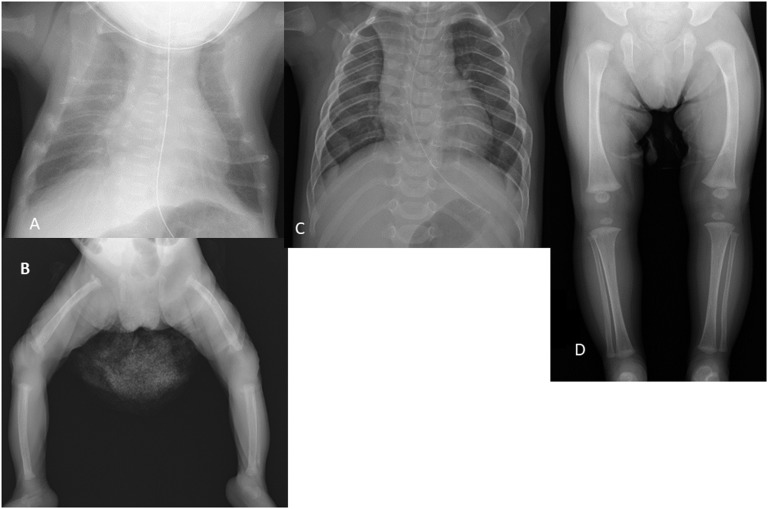
X-ray findings of the patient. (A, B) Neonate. Osteopenia of the skeleton is observed. The ribs are thin and wavy, the thorax is bell shaped and narrow, and the cortex of the rims is coarse, thin, and markedly demineralized. The femora are mildly bowed, and metaphyseal constriction resulting from fracture is observed (arrow). (C, D) Six months of age. The skeletal lesions almost completely resolved.

A female baby was born at 37 weeks’ gestational age to a 29-year-old healthy Japanese woman by cesarean section because of pelvic position and twin pregnancy. Her birth weight was 2140 g (−1.6 SD) with birth length of 45.0 cm (−1.3 SD). Apgar scores were 4 at 1 minute and 8 at 5 minutes. Shortly after birth, the infant was hospitalized in the neonatal intensive care unit at our hospital because of respiratory distress. Among her physical findings on admission, tachypneic and subcostal retractions were observed. Chest-abdominal X-ray images revealed thoracic deformity and skeletal osteopenia (Fig. 1). Subperiosteal resorption of cortical bones and coarse trabecular bones were observed, the appearance of which matches the skeletal findings of hyperparathyroidism. Her twin brother’s perinatal course was uneventful. His birth weight was 2345g (−1.2 SD). He showed mild PTH elevation and low 25-hydroxy vitamin D, but no abnormal skeletal finding. Maternal calcium and phosphate were within normal ranges, but vitamin D deficiency was found, along with a high PTH level for the lactation period (Table 1). Initially, we diagnosed the patient as having secondary hyperparathyroidism caused by maternal vitamin D deficiency; however, reasons for the discordance in clinical symptoms between the twin siblings were unclear at that time. We administered vitamin D analog (alfacalcidol) and calcium lactate to the patient. After treatment, the PTH level decreased gradually. It had normalized by 6 weeks of age (Table 2). Bone mineral content (BMC) and areal bone mineral density (BMD) of her lumbar spine (L2-L4) were measured using dual X-ray absorptiometry (Lunar Prodigy, GE Healthcare) at 2 months of age. Her BMC was 0.50 g; BMD was 0.101 g/cm^2^ (*z* score, −6.97 SD). Those values were remarkably lower than those of age-matched Canadian children [5]. Respiratory support was required up to 2 months of age. By 4 months, her osteopenia had gradually improved: BMC and BMD were 1.05 g and 0.164 g/cm^2^, respectively. She was discharged from the neonatal intensive care unit with enteral tube feeding. By 6 months of age, X-ray images showed that her skeletal deformity had resolved almost completely (Fig. 1). At 11 months of age, she completed her treatment with alfacalcidol. On review at age 18 months, she showed catch-up in growth and developmental milestones, with no recurrence of skeletal deformity occurred after she completed the treatment.

**Table 1.** Laboratory Findings for the Patient, Twin Sibling, and Mother

	Patient (Day 0)	Twin Sibling (Day 4)	Mother (Day 5 Postpartum)	Normal Range
Intact PTH, pg/mL	2700	176	56	14–72
Alkaline phosphatase, U/L	998	887	409	Adult: 106–322
				Neonate: 530–1610
Calcium, mg/dL	7.7	8.3	8.2	Adult: 8.8–10.1
				Neonate: 9.0–11.2
Phosphate, mg/dL	5.6	6.8	3.3	Adult: 2.7–4.6
				Neonate: 4.6–8.0
25(OH)D, ng/mL	9	5	6	20–50
Albumin, mg/dL	3.1	3.7	3.0	Adult: 3.8–5.3
				Neonate: 3.0–4.1
Creatinine, mg/dL	0.59	0.53	0.44	0.46–0.79
Urinary calcium/creatinine ratio	0.045	Undetectable	Undetectable	<0.14

Abbreviation: 25(OH)D, 25-hydroxy vitamin D.

**Table 2.** Course of Clinical Laboratory Data in the Patient

Age	At Birth	2 Wk	4 Wk	6 Wk	3 Mo	9 Mo	15 Mo	Normal Range
Intact PTH, pg/mL	2700	1257	99	70	13	17	35	14–72
Alkaline phosphatase, U/L	998	2639	1847	1648	1715	1235	1110	Neonate: 530–1610
								1–2 y: 395–1289
Calcium, mg/dL	7.7	9.3	9.4	9.8	9.9	10.2	10.0	Neonate: 9.0–11.2
								1–2 y: 8.8–10.6
Phosphate, mg/dL	5.6	3.7	5.5	5.6	5.3	5.7	4.8	Neonate: 4.6–8.0
								1–2 y: 3.9–6.2
25(OH)D, ng/mL	9			39	45	17	15	20–50

## 2. Subjects and Methods

The study was approved by the Institutional Ethical Review Board of the University of Miyazaki. Written informed consent was obtained from legal guardians. Mutation analysis of the *TPRV6* gene in peripheral blood lymphocyte by Sanger sequencing was conducted on the patient, her twin brother, and their parents. Information related to PCR primers and conditions is available upon request.

## 3. Results

We identified compound-heterozygous mutations in the *TRPV6* gene; the patient had c.668T>C, (p.Ile223Thr) and c.1282G>A (p.Gly428Arg). The allele with c.668T>C was inherited from her father; c.1282G>A was from her mother. These missense mutations were reported earlier as pathological mutations [3]. Neither mutation was detected in her twin sibling.

## 4. Discussion

One of the dizygous twin siblings presented with TNHP with severe skeletal undermineralization and respiratory distress. The twin sibling in the same uterine environment showed no pathological symptoms. Genetic analysis clarified the *TRPV6* gene variant in this patient with severe clinical manifestation. The twin sibling demonstrated that maternal vitamin D deficiency does not affect the fetal phenotype. Calcium metabolism of the fetus is regulated differently than that of a neonate. A human fetus typically accumulates 30 g of calcium by term through the placenta. The Ca^2+^ concentration in the fetus is set markedly higher than the maternal level to sustain adequate mineralization of the fetal skeleton [6, 7]. Multiple animal and human studies have shown that maternal vitamin D deficiency and genetic absence of the vitamin D receptor or calcitriol do not affect fetal calcium [6]. The existence of maternal–fetal active Ca^2+^ transport through the placenta has been suggested to maintain higher Ca^2+^ concentration in the fetus than in maternal circulation, but details of the related molecular mechanisms have remained unclear until recently. In fact, TRPV6 plays an important role in the apical Ca^2+^ entry pathway for intestinal Ca^2+^ absorption [2]. TRPV6 is expressed mainly in intestines, but it is also expressed in placental trophoblast [1, 8]. In *Trpv6* knockout (KO) mice, the transport activity of radioactive Ca^2+^ from mother to fetus was found to be 40% lower than in wild-type mice. The ash weight in *Trpv6* KO mice is also lower than in wild-type mice [1]. Despite the strong proof of important roles in calcium homeostasis, TRPV6 was not implicated in human disease in any study reported during the past decade. Recently, Suzuki *et al.* [3] reported that inactivating *TRPV6* gene mutation resulted in TNHP with severe undermineralization. They found that TRPV6 variants interfered with the placental maternal–fetal Ca^2+^ transport from functional analysis. The *TRPV6* variants engender fetal hypocalcemia, secondary hyperparathyroidism, and skeletal demineralization because of the combined effects of impaired primary mineralization and increased bone resorption. To date, seven cases of TNHP caused by *TRPV6* recessive mutations have been reported. All subjects of those studies presented hypoplastic thorax and postnatal respiratory distress [3, 4]. It is particularly interesting that vitamin D deficiency was coincident in most reported cases with *TRPV6* variant, as it was in our case. TRPV6 expression is regulated strongly by 1,25-dihydroxy vitamin D [9]. We hypothesized that TNHP caused by the *TRPV6* variant develops with the existence of both *TRPV6* impairment as a genetic factor and vitamin D deficiency as an environmental factor.

After delivery, the main calcium source changes dramatically from the placenta to intestinal absorption. In general, epithelial Ca^2+^ transport in intestines has two main pathways: transcellular and paracellular [2]. The process of transcellular pathway is Ca^2+^ transport by crossing of both apical and basolateral membranes of the epithelial cells. By contrast, the paracellular pathway is simple diffusion and passive transport through the tight junctions. The paracellular pathway is the predominant form of Ca^2+^ absorption in intestines under physical conditions [10]. Despite insufficient Ca^2+^ supply *in utero* because of impaired *TRPV6*, when adequate nutrition is provided from the diet after birth, transport of Ca^2+^ from the intestines is secured by the paracellular pathway; therefore, clinical symptoms of *TRPV6* variant resolved during infancy.

In summary, we describe a case of TNHP caused by *TRPV6* gene mutations. TNHP with *TRPV6* gene variants is a unique disease in terms of its transient pathology *in utero* and relief after birth.

## Abbreviations:

BMC: bone mineral content
BMD: bone mineral density
Ca2+: calcium
KO: knockout
TNHP: transient neonatal hyperparathyroidism
TRPV6: transient receptor potential subfamily V, member 6
